# 

**Published:** 1827-04

**Authors:** 


### Contents

OF

No. 12. Vol. VI. (NEW SERIES) for APRIL, 1827.

[*SECOND NUMBER FOR* 1827.]

Art. I.

Propositions or Principles of Medicine. By F. S. V. Broussais, M.D. &c.

Part 1. Physiology, or the Laws of Health .......................... 314

Part 2. Pathology, or the Laws of Disease .......................... 321

II.

Medico-Chirurgical Transactions, Vol. XIII. concluded.
1. Dr. Thomson on Hydrometra, and Dry Gangrene .......................... 340
2. Dr. Marshall Hall and Mr. Higginbottom on a destructive Inflammation of the Eye .......................... 342
3. Dr. Johnson on a remarkable Disease of the Heart.......................... 343
4. Dr. Elliotson on Subcarbonate of Iron .......................... 345
5. Dr. Gregory on Hydrophobia .......................... 347

III.

CHLORURETS (CHLORIDES) OF SODA AND OF LIME.

1. Mr. Scott's Translation of Labarraque's Work..........................

2. Mr. Alcock's Essay on the Use of Chloruret (Chloride) of Soda and of Lime, as disinfecting Agents .......................... } 349

IV.

Researches on the Paralysis attendant on Insanity .......................... 362

V.

Observations on the Surgical Pathology of the Larynx and Trachea. By W. H. Porter, A. M. &c. .......................... 375

VI.

Dr. Whitelaw Ainslie on the Materia Indica, &c.......................... 397

VII.

Dr. L. Martinet's Manual of Pathology. By Mr. Quain.......................... 407

VIII.

Mr. Charles Bell's Consultations and Cases illustrative of his Papers on the Nerves .......................... 412

IX.

A Dissertation on Local Affections of the Nerves, drawn up under the Inspection of M. Beclard; with communications from Dupuytren, Marjolin, Richerand, Roux, &c. By M. Jul. Descot.......................... 424

X.

Physiological Enquiry respecting the Action of Moxa, in various Diseases. By William Wallace, M. R. I. A. &c. .......................... 448

### XI.

### Quarterly Periscope

OF

### PRACTICAL MEDICINE.

### PART I.

1. M. Dufau on Intermittent Irritations .......... 457
2. On Phlegmasia Dolens .......... 464
3. Case of M. Talma, with the Dissection .......... 465
Case of the late General Kyd, with the Dissection .......... 466
4. M. Piorry on Viper Bite, with the Application of the Cupping-glass .. 468
5. Dr. O'Connor and Dr. Fergusson on the Bische, a malignant Species of Dysentery .......... 470
6. Fatal Case of Abdominal Injury from a Fall, with Dissection .......... 472
7. Messrs. Rigot and Trousseau on Post-mortem Changes in the Mucous Membranes .......... 474
8. Mr. Wood on Disease of the Thymus Gland, and Sudden Death .......... 477
9. Instance of Fatal Mortification of the Great Toe .......... 480
10. Dr. Monro on Obliterations of the Aorta .......... 481
11. Homicide—Suicide—Infanticide, &c.
1. Remarkable Case and Trial of Harriet Cornier .......... 482
2. Case of Parricide .......... 486
3. Case of Mons. Tristel—poisoning .......... 489
4. Dreadful Case of Matricide .......... 490
5. Case of Infanticide .......... 491
6. Remarkable Case of Homicide .......... 492
12. On Chronic Ulcers of the Stomach .......... 493
13. On Dysmenorrhœa, by Dr. Campbell .......... 494
14. Remarkable Instance of Extra-uterine Fœtus .......... 496
15. M. Piorry's Pleximetre .......... 496
16. Case of Intestinal Ulceration and Hæmorrhage .......... 497
17. Mr. Thorn on Extract of Balsam of Copaiba .......... 499
18. Carbonate of Iron in Neuralgic Affections .......... 500
19. Cases of General Vascular Inflammation .......... 501
20. On Purgation as a Remedy in Diseases .......... 504
21. Cases of Epilepsy, cured by Digitalis, and by Purgatives .......... 508
22. Experiments on Digestion in the living Human Stomach .......... 510
23. An Essay and Experiments on White Mustard Seed .......... 512
24. M. Andral on Alterations in the State of the Bile .......... 515
25. Mr. Haslam on the Secale Cornutum .......... 517
26. Dr. Hastings on Mollescence of the Lungs .......... 517
27. Death after the Removal of a Testicle .......... 520

### Quarterly Periscope

OF

### PRACTICAL MEDICINE.

### PART II. HOSPITAL REPORTS.

1. M. Velpeau on Pleurisy and Abscesses succeeding Surgical Operations—*(Hospice de Perfectionnement.)*

Part 1. On Pleurisy after Operations and Wounds .......... 521

Part 2. On Tubercular Abscesses, chiefly in the Liver, after Surgical Operati ns, and extensive Suppurations .......... 525

2. On Injuries of the Head—*(Middlesex Hospital.)* .................... 529
3. Hospital Report of the HOTEL DIEU, (Autumnal Quarter.)
1. On Intermittents, treated in various ways.................... 531
2. On Arachnitis.................... 532
3. On Paralysis .................... 532
4. On Prussic Acid.................... 532
5. On Hæmoptysis .................... 533
6. On Angina Pectoris .................... 533
7. On Intestinal Ulceration after Fevers.................... 534
4. Mr. Earle on Cellular Inflammation—*(Bartholomew's)*.................... 535
Report on Incisions in this Disease in the Year 1813, by Dr. Johnson, 536
Report of a Case in St. Thomas's Hospital, treated by Incisions...... 537
5. M. Lisfranc on Ulcers, simple and varicose—*(La Pitié.)*.................... 538
Effects of the Chlorides of Lime and Soda in Ulcers.................... 539
6. Report on Injuries of the Head—*(Middlesex Hospital.)* .................... 541
7. M. Dupuytren on Congenital Dislocation of the Hip-joint—*(Hôtel Dieu)* 544
8. Mr. Shaw on Dissection Wounds—*(Middlesex)*.................... 546
9. Aneurism from Venesection—*(St. George's Hospital)* .................... 547
10. Surgical Reports of M. M. Roux, Breschet, and Dupuytren *(Hosp. de la Faculté)* .................... 548
Stone in the Bladder—various Modes of Lithotomy, &c.................... 549
11. M. Lisfranc on Cancer of the Penis—*(La Pitié.)* .................... 556
12. Professor Mackenzie on Rheumatic Ophthalmia—*(Glasg. Eye Infirm.)* 558
13. Reports of M. M. Richerand and Cloquet—*(Saint Louis.)*
1. Operation for Hernia, wounded Intestine, Entero-raphia .................... 560
2. On the Use of Nitrate of Quicksilver .................... 561
3. Acetate of Lead, as a Sedative in Cancerous Diseases.................... 561
4. Cranial Fractures, with Cases .................... 561
14. Mr. Jeffreys on Constitutional Disposition to Scirrhus—*(St. George's)* 562
15. M. Lisfranc on Extirpation of the Parotid Gland—*(La Pitié.)* ...... 564
16. Dr. Mac Andrew on Induration of the Cellular Tissue—*(South London Dispensary)*.................... 565
17. On the Deuto-Iodine of Mercury—*(Saint Louis)* .................... 566
18. Amaurosis cured by Vomiting—*(Hospital of Florence)*.................... 568

### Quarterly Periscope

OF

### PRACTICAL MEDICINE.

PART III. ANALECTA.

1. On the Division of Medicine into Physic and Surgery .................... 569

2. Remarkable Case of Ileus from Calculous Concretions.................... 574

3. Case of Syncope Anginosa .................... 574

4. On Transfusion in Uterine Hæmorrhage.................... 575

5. Retention of Urine—false Passage—Death .................... 576

6. Sciatica cured by Ol. Terebinthinæ .................... 577

Palpitation suspended by Carbonate of Iron .................... 578

7. On Axillary Aneurism—Mr. Liston's Operation .................... 579

8. On Clinical Medicine, &c. Dr. Clark .................... 580

On the Inefficacy of Medical Examinations .................... 583

9. Dr. Bellingheri on Duodenitis .................... 585

10. Hypertrophy of the Heart as a Cause of Apoplexy.................... 586

11. On Regeneration of a sphacelated Urethra—Answer to Dr. Brown.... 589

12. Remarkable Disease of the Stomach.................... 590

13. Cephalo-spinal Fluid .................... 591

14. Dr. Thomson on the Treatment of Acute Rheumatism, with Remarks, 593
15. Phlebitis—Consecutive Œdema—Phlegmatia Dolens ............... 595
16. Three Cases of Hernia of the Stomach through the Diaphragm...... 599
17. Remittent Broncho-Encephalitis ................................ 600

EXTRA-LIMITES.

I.

Prize Hospital Report, from St. Thomas's Hospital.

(Prize adjudged to Mr. Thomas H. Smith, Pupil.)

No. 1.

I. Cases of Fracture of the Spine, with Operations, &c. By Mr. Tyrrell ................................................ 601
II. Case of Venereal Lepra, following Gonorrhœa and Superficial Ulceration................................................ 606
III. Cases of Intermittent Fever, variously treated, with Remarks .. 606
IV. Two Cases of Strangulated Hernia, with unusual Phenomena.... 609
V. Case of Hooping Cough, with the Appearances on Dissection .... 612
Case of lateral partial Dislocation of the Cervical Vertebræ.... 613
VI. Case of Traumatic Tetanus, the Post-mortem Examination, and Abstract of Mr. Tyrrell's Clinical Lecture on Tetanus ...... 614
VII. Operations above the Knee ........................................ 617
Lithotomy ........................................ 618
Popliteal Aneurism........................................ 619
Amputation ........................................ 619
II. The Edinburgh Reviewer's Reply to Dr. Barry ..................... 620
III. Mr. Jowett's Case of Dropsy of the Pericardium .................. 623
VI. New Medical Doctrine of Italy........................................ 626
V. Dr. Granville on the disinfecting Liquid of Soda .................. 629
VI. Plan of the Academy of Minute Anatomy .......................... 630

XII.

Bibliographical Record ........................................ 632

XIII.

INTELLIGENCE, &c.

1. Notice respecting the Prize Hospital Reports ...................... 635
2. Chlorides of Soda and Lime ........................................ 636
3. Medico-Chirurgical Society.—Address of the new President, Mr. Travers........................................ 636
4. Circular respecting the Admission of Licentiates to the Hunterian Museum ........................................ 636
5. Obituary of the late Mr. Bampfield, with the Nature of his Disease.. 636
6. Works published by M. Bailliere ........................................ 637
7. Answer to Dr. Barnes, of Carlisle ........................................ 638
8. Mr. Lizars to his Subscribers........................................ 638
9. Suggestion of a Correspondent respecting Cooper's Surgical Dictionary 638
10. "Sound Chirurgical Knowledge," or a correct statement of facts respecting the case of Aneurism from Venesection, at the Westminster Hospital ........................................ 638

List of Subscribers ........................................ 640

